# Synoviocytes from pigmented villonodular synovitis are less sensitive to cadmium-induced cell death than synoviocytes from rheumatoid arthritis

**DOI:** 10.1038/s41598-022-07745-9

**Published:** 2022-03-09

**Authors:** Héléna Farese, Mélissa Noack, Pierre Miossec

**Affiliations:** Immunogenomics and Inflammation Research Unit, Edouard Herriot Hospital, Hospices Civils de Lyon, 5 Place d’Arsonval, 69003 Lyon, France

**Keywords:** Immunology, Biogeochemistry, Rheumatology

## Abstract

Pigmented villonodular synovitis (PVNS) is a rare inflammatory articular disease sharing common characteristics with rheumatoid arthritis (RA), notably hyperplasia of the synovium due to a hyperproliferation of synoviocytes, and with cancer owing to mutations of the CSF1/M-CCSF gene. Targeting synovium hyperplasia by the local delivery of Cadmium (Cd) has been already tested in vitro and in vivo models of RA and could be applied to PVNS. PVNS and RA synoviocytes were exposed to low doses of Cd. After different culture time points, a qualitative analysis was done by microscopy and quantitative measurements of apoptosis, cell viability and IL-6 production were carried. IL-6 production by PVNS synovial tissue was also quantified after Cd treatment with or without the presence of pro-inflammatory cytokines (IL-17 + TNF). Addition of Cd induced cell death in both PVNS (1 ppm) and RA (0.1 ppm) synoviocytes, which increased with time and Cd concentrations. Cd increased the percentage of apoptotic cells and decreased cell viability and IL-6 production. In all these experiments, PVNS synoviocytes were tenfold less sensitive to Cd than RA synoviocytes. Cd decreased IL-6 production by PVNS synovial tissue and its effect was enhanced with pro-inflammatory cytokines. In summary, PVNS synoviocytes show resistance to Cd-induced cell death and decreased inflammation. Intra-articular use of Cd could represent a potential therapeutic tool in PVNS.

## Introduction

Described for the first time in 1941, pigmented villonodular synovitis (PVNS), also known as tenosynovial giant cell tumor, is a rare articular disease characterized by an inflammatory synovitis with proliferation of synoviocytes and accumulation of large size monocyte-derived osteoclasts in the synovial tissue of joints^1^. Affecting mostly a single joint, PVNS usually affects young adults with an annual incidence of 1.8 cases out of one million^2,3^. The wide spectrum of clinical manifestations includes mainly pain, inflammation and joint swelling leading to a limitation of joint function^4^. Synovium hyperplasia is one of the critical hallmarks leading to an aggressive synovitis causing rapid joint degradation through cartilage and bone destruction^5^.

The disease combines features of a neoplastic disease and of an inflammatory disease, with similarities to rheumatoid arthritis (RA)^6^. The molecular changes combine a specific translocation of the monocyte colony-stimulating factor 1 (M-CSF1) gene that leads to CSF1/M-CSF overexpression resulting in an accumulation of non-neoplastic monocyte-like inflammatory cells and inflammatory cytokine production as in RA.

For many years, its main treatment was based on open and arthroscopic surgery but remains associated with a high risk of recurrence. More recently, the use of CSF1 receptor (CSF1R) inhibitors such as imatinib, nilotinib or pexidartinib elicited antitumor activity in patients with PVNS^7,8^. However, severe adverse events have limited the use of these systemic treatments to a small number of patients when surgery is not possible. Thus, there remains a need of new local therapies^9,10^.

The lack of new local treatments apart from surgery creates the need to identify new options for the local treatment of arthritis. In this context, targeting of stromal cells/synoviocytes with metals such as Cadmium (Cd) needs to be evaluated^11^. Intra-articular administration may represent an interesting advantage to limit systemic toxicity. In preclinical studies linked to RA, the use of Cd has demonstrated the induction of massive cell death in RA synoviocytes but not in immune cells in vitro*,* and protection against joint destruction in vivo in a rat RA animal model^8,12^. With this clear anti-proliferative and anti-inflammatory potential in the context of RA, it could be interesting to apply Cd therapy to PVNS.

Based on these previous results and on the shared characteristics between PVNS and RA, this study aimed to test in vitro the effect of low dose Cd exposure in the context of PVNS and to compare its effect on synoviocytes from RA and PVNS patients.

## Results

### A similar Cd-induced cell death requires higher concentrations of Cd in PVNS compared to RA synoviocytes

The effect of Cd exposure was first evaluated qualitatively by light microscopy. PVNS and RA synoviocytes were cultured in the presence or absence (control) of Cd at different concentrations (0.01, 0.1, 1 and 10 ppm) and different time points (24 h, 72 h and 6 days) to evaluate a possible dose- and time-effect.

**Figure 1:**
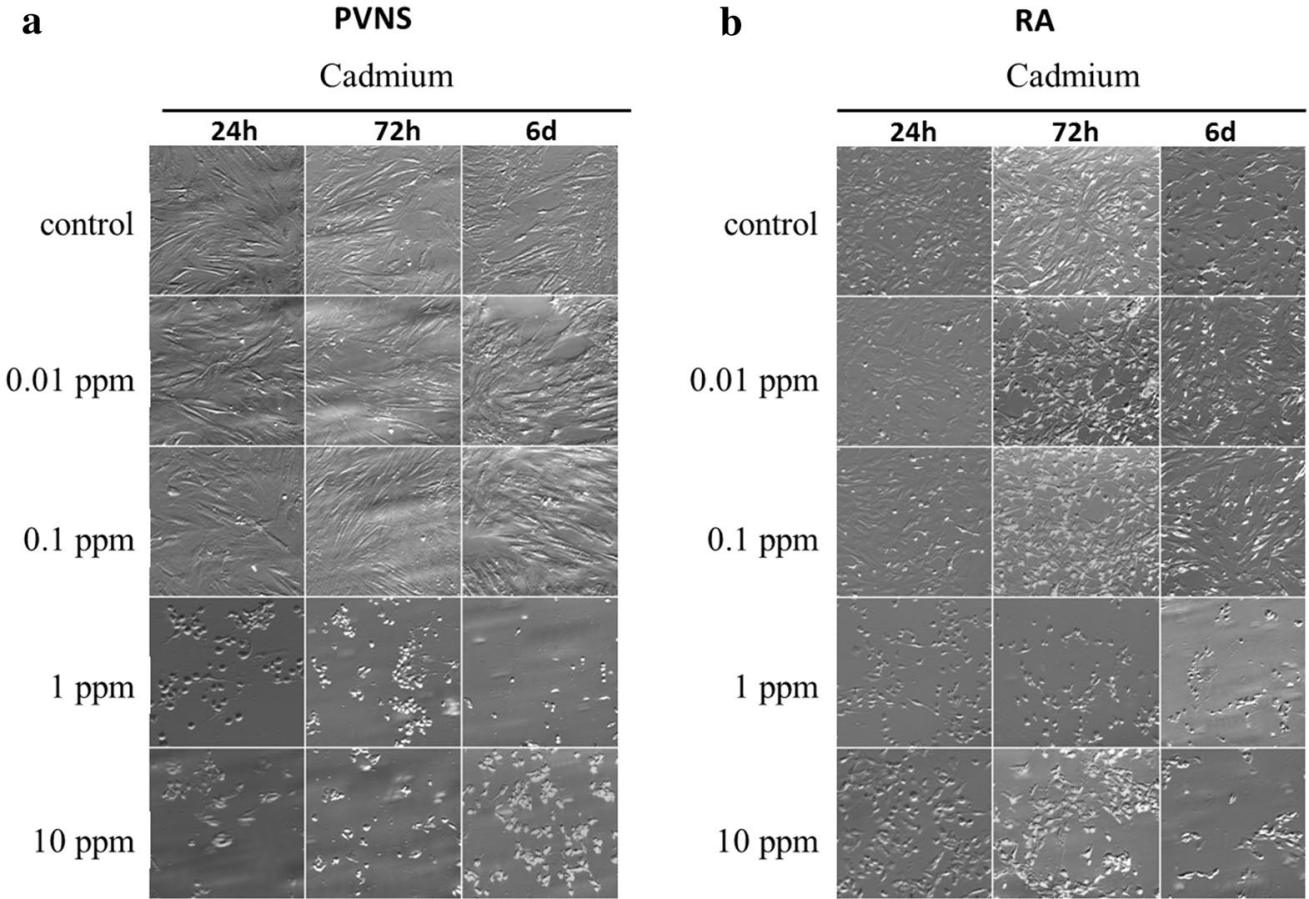
Observation of cell death by light microscopy. PVNS (**a**) and RA (**b**) synoviocytes, used between passages 4 to 9, were treated with HNO_3_ as the control or with increasing concentrations of Cd (0.01 ppm; 0.1 ppm; 1 ppm and 10 ppm) to quantify a dose–response effect. Synoviocytes were observed by optic microscopy following a kinetic at 24 h, 72 h and 6 days after the Cd exposure to evaluate a possible time effect.

In the control condition, after 24 h of culture, PVNS synoviocytes were adherent with a fibroblast-like morphology, and had an outspread aspect that remained unchanged with time. Cd at 0.01 and 0.1 ppm had no effect, as cell morphology was like the control condition with no time-effect (Fig. 1a). At 1 and 10 ppm, Cd induced cell death from 24 h (Fig. 1a). The morphology of synoviocytes was modified with a round shape and floating cells compared to the fibroblast-like morphology described in the control condition. This Cd effect was increased after 72 h and up to 6 days of exposure with a complete lethal effect at 10 ppm, where no remaining adherent cells were seen. Moreover, an exposure-time effect was observed as cell death increased with the exposure duration (Fig. 1a).

In comparison, Cd treatment in RA synoviocytes induced the same effect as in PVNS cells but starting at a lower concentration (Fig. 1b). At 24 h, cell death morphology, with a round shape, appeared from 0.1 ppm of Cd compared to 1 ppm for PVNS cells (Fig. 1a,b, respectively). Furthermore, cell death increased with Cd concentration showing a dose–response effect. At 72 h and at 6 days, cell death occurred using the lowest concentration of 0.01 ppm increasing up to 10 ppm of Cd where a complete lethal effect was observed (Fig. 1b). As for PVNS cells, a time–effect was observed.

These observations showed a cell death effect of Cd with a dose–response for both cell types. However, the same effect was elicited at a higher concentration of Cd for PVNS compared to RA synoviocytes. These results showed that PVNS synoviocytes were approximately tenfold less sensitive to Cd-induced cell death than RA synoviocytes.

### A higher concentration of Cd is needed to reduce cell viability in PVNS vs. RA synoviocytes

After a qualitative observation, the aim was to quantify the cell viability of PVNS and RA Cd-exposed synoviocytes. Based on the above microscopy results, PVNS and RA synoviocytes were exposed to Cd, for 6 days to obtain a maximal effect, at the same concentrations (0.01, 0.1, 1 and 10 ppm). After 6 days of culture, a neutral red assay was performed to quantify cell viability. The observation by light microscopy in the control condition has permitted to define a threshold of cell viability. As no cell death was observed in this condition for both PVNS and RA synoviocytes, 100% of viability in the control condition was used as a reference.

**Figure 2:**
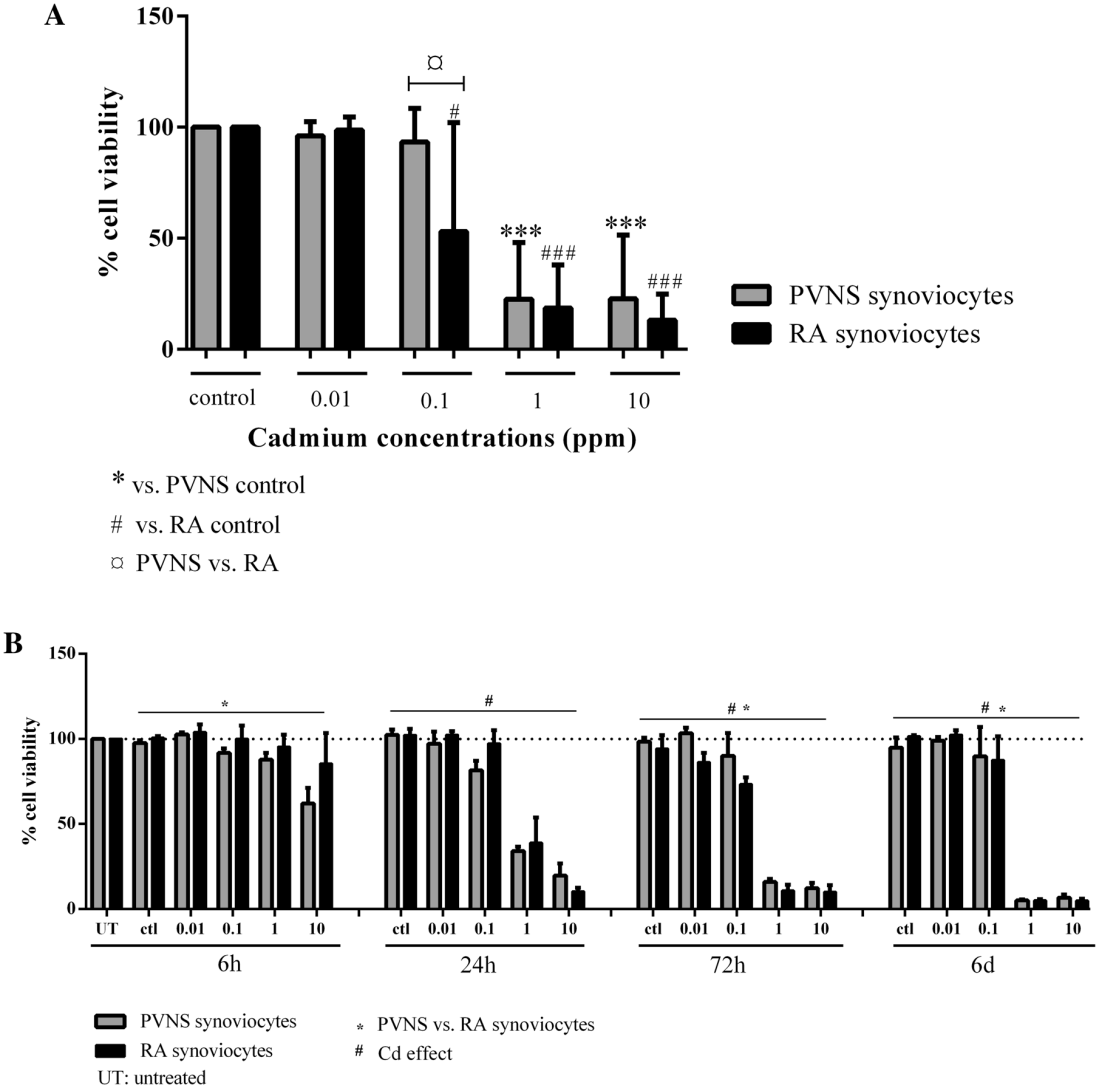
Quantification of cell viability of PVNS and RA synoviocytes 6 days after Cd exposure. PVNS and RA synoviocytes, used between passages 4 to 9, were treated with increasing concentrations of Cd (0.01 ppm; 0.1 ppm; 1 ppm and 10 ppm; (**A**), n = 12 from 6 PVNS patients and 6 RA patients) and, in independent experiments, following kinetics (6 h, 24 h, 72 h and 6 days; (**B**), n = 3 from 3 PVNS patients and 3 RA patients). A quantification of cell viability was done by neutral red assay 6 days after Cd exposure of synoviocytes (**A**) or following kinetics (**B**). Results are presented as mean ± SD, *p < 0.05.

The lowest dose of Cd (0.01 ppm) did not induce cell death in PVNS and RA synoviocytes (96.0 ± 6.4% vs. 98.7 ± 5.8%, respectively; Fig. 2A). The cell death effect of Cd started at the concentration of 0.1 ppm in RA synoviocytes with a decrease of cell viability compared to control (52.9 ± 49.2% vs. 100% for control, p = 0.031, Fig. 2A); while at the same concentration of Cd, PVNS synoviocytes were not affected (93.3 ± 15.1%, Fig. 2A). Similarly to the results from microscopy, at 1 and 10 ppm of Cd, the percentage of viability in both PVNS and RA cells was less than 25%, confirming a massive cytotoxic effect of Cd (1 ppm: 22.5 ± 25.6%; 10 ppm: 22.8 ± 28.6% for PVNS cells, p = 0.0005, and 1 ppm: 18.6 ± 19.3%; 10 ppm: 13.1 ± 11.7% for RA cells, p = 0.0005, Fig. 2A).

In addition, kinetic studies were performed to extend the microscopy results. Results were analyzed using a two-way Anova test. Starting at 6 h, the presence of Cd reduced cell viability and this effect became significant at 24 h (6 h: p = 0.09; 24 h, 72 h and 6 days: p < 0.0001; Fig. 2B). At 6 h, PVNS cells were more sensitive to cell death than RA synoviocytes (p = 0.009; Fig. 2B) but at 72 h, RA synoviocytes displayed a lower percentage of cell viability, mainly at 1 and 10 ppm (p = 0.01, Fig. 2B). These results confirmed the Cd-dose and -time effect.

Such results constituted a first analysis demonstrating quantitatively the cytotoxic effect of Cd in both PVNS and RA synoviocytes and highlighting the resistance of PVNS compared to RA synoviocytes to Cd-induced cell death.

### Cd exposure induces apoptosis in PVNS and RA synoviocytes

To explore if cell death induced by Cd was through apoptosis, synoviocytes were analyzed by Annexin V staining. As described above, PVNS and RA synoviocytes were exposed for 6 days to increasing Cd concentrations (0.01, 0.1, 1 and 10 ppm).

**Figure 3:**
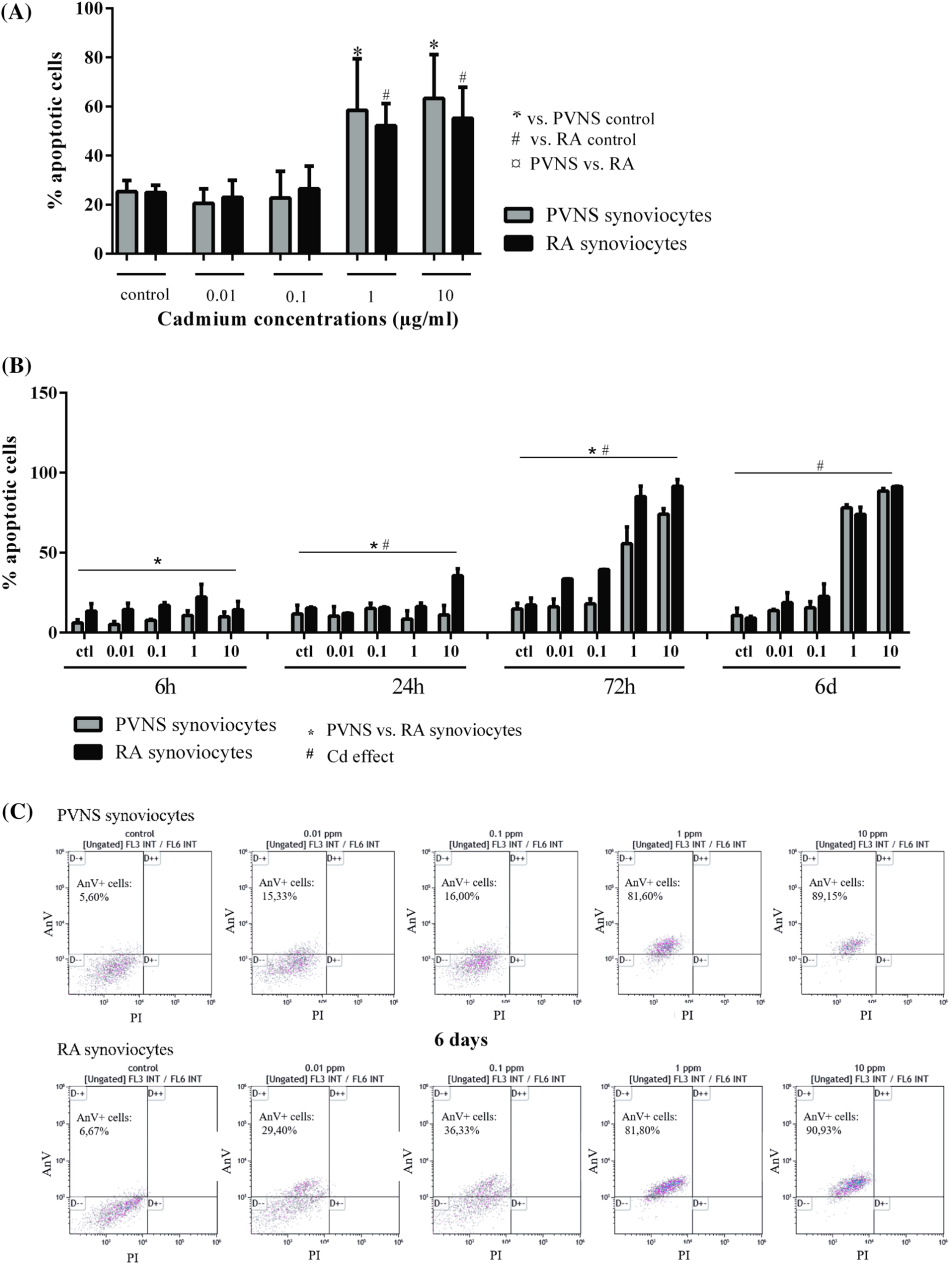
Quantification of apoptotic cells in cultures of PVNS and RA synoviocytes 6 days after Cd exposure. After 6 days (**A**, n = 5 from 5 PVNS patients and 5 RA patients) of treatment with Cd (0.01 ppm; 0.1 ppm; 1 ppm and 10 ppm) or in independent experiments, following kinetics (6 h, 24 h, 72 h and 6 days; **B**, n = 3 from 3 PVNS patients and 3 RA patients), Annexin V staining of PVNS and RA synoviocytes, used between passages 4 to 9, was done to evaluate if cell death induced by Cd is mediated through apoptosis. Results are presented as mean ± SD, *p < 0.05. (**C**) The FACS plots obtained for cells from one PVNS and one RA patient, at day 6, for the different concentrations of Cd.

The basal percentage of apoptotic cells in the control condition was similar for both PVNS and RA synoviocytes (25.3 ± 4.5% vs. 25.0 ± 3.0% respectively, Fig. 3A). Moreover, the percentage of apoptotic cells did not change after the exposure to low-doses of Cd, 0.01 and 0.1 ppm, for both PVNS and RA synoviocytes (PVNS: 0.01 ppm: 20.5 ± 5.9%; 0.1 ppm: 22.7.0 ± 10.9% vs. 25.3 ± 4.5% for control; RA: 0.01 ppm: 23.0 ± 7.0%; 0.1 ppm: 26.5 ± 9.2% vs. 25.0 ± 3.0% for control, Fig. 3A), while 0.1 ppm of Cd already induced a decreased cell viability in RA synoviocytes (Fig. 2). At 1 ppm of Cd, a strong increase of 50–60% of apoptotic cells was detected for both PVNS and RA synoviocytes (58.5 ± 21.0% and 52.2 ± 8.9% respectively, p = 0.031, Fig. 3A). The percentage of apoptotic cells continued to slightly increase with 10 ppm for both cell types (63.3 ± 8.0% for PVNS and 55.2 ± 5.7% for RA, p < 0.05, Fig. 3A).

As for cell viability, a kinetic experiment was performed to follow apoptotic cells over time (6 h, 24 h, 72 h and 6 days; Fig. 3B) and a two-way ANOVA test was performed to compare the effect of cell origin (PVNS vs. RA) and of Cd. At 6 h, no Cd effect was detected, but RA synoviocytes showed a higher percentage of apoptotic cells than PVNS synoviocytes (p = 0.004; Fig. 3B). This difference between RA and PVNS cells was maintained at 24 h and 72 h (24 h: p = 0.02; 72 h: p = 0.001, Fig. 3B) but was lost at 6 days. The Cd effect was significant starting at 24 h (p = 0.04) and increased with time (24 h and 72 h: p < 0.0001, Fig. 3B). At 6 days, almost all synoviocytes from both PVNS and RA were positive for Annexin-V, for 1 and 10 ppm of Cd. These results were in line with the cell viability results at 72 h and 6 days. The discrepancy at 24 h between cell viability and apoptosis may suggest that at early stages, a mechanism other than apoptosis is involved. In Fig. 3C, at 6 days, the increase of apoptosis was linked to the increase of Cd concentration.

These results confirmed that the Cd-induced cell death in PVNS and RA synoviocytes was mediated, at least in part, through apoptosis.

### Cd exposure induces a decrease in IL-6 production by PVNS and RA synoviocytes

To quantify the effect of Cd on inflammation, the production of IL-6 was measured in cell culture supernatants. This cytokine is associated with the physiopathology of PVNS and RA, with high levels in synovial fluid of patients with both diseases^13,14^. As described above, PVNS and RA synoviocytes were treated with different concentrations of Cd (0.01, 0.1, 1 and 10 ppm) and after 48 h of culture, IL-6 levels were measured in the supernatants.

**Figure 4:**
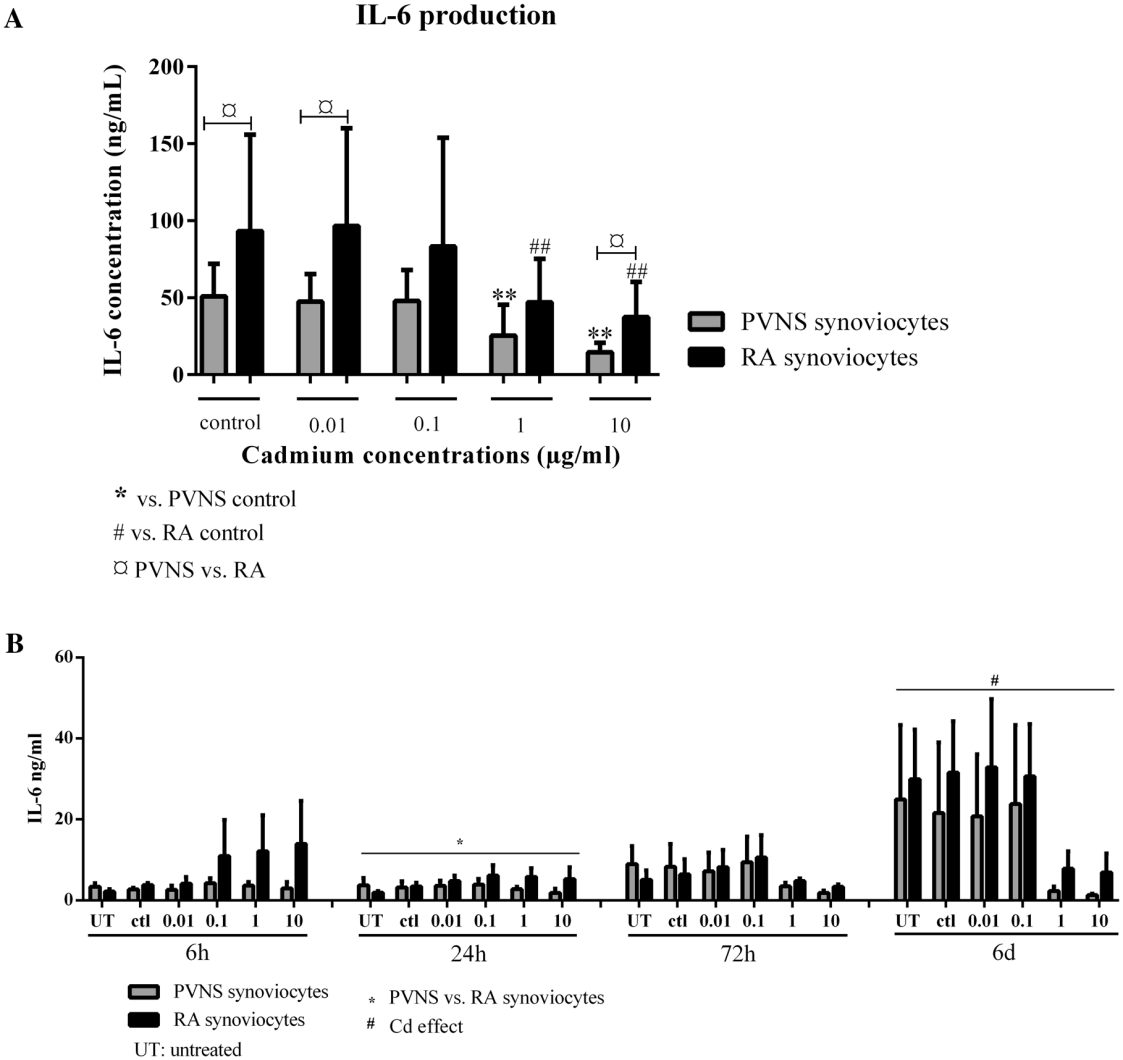
IL-6 production in PVNS and RA synoviocyte supernatants 48 h after Cd exposure. Supernatants from PVNS and RA synoviocytes, used between passages 4 to 9, were collected 48 h after the treatment with increasing concentrations of Cd (0.01 ppm; 0.1 ppm; 1 ppm and 10 ppm; (**A**), n = 10 from 5 PVNS patients and 5 RA patients) or, in independent experiments, following a kinetic study (6 h, 24 h, 72 h and 6 days; (**B**); n = 3 from 3 PVNS patients and 3 RA patients). A quantification of IL-6 production was done by ELISA. Results are presented as mean ± SD, *p < 0.05.

In PVNS, Cd exposure with concentrations of 0.01 and 0.1 ppm had no effect on IL-6 production compared to the control (47.5 ± 17.9 and 47.9 ± 20.1 ng/ml, respectively, vs. 50.9 ± 21.1 ng/ml, Fig. 4A). At 1 ppm, IL-6 production was strongly decreased compared to the control (25.4 ± 20.1 ng/ml vs. 50.9 ± 21.1 ng/ml, p = 0.005, Fig. 4), and even more so at 10 ppm (14.6 ± 6.1 ng/ml vs. 50.9 ± 21.1 ng/ml, p = 0.002, Fig. 4A).

In RA, IL-6 production in the control condition was higher than that of PVNS (93.3 ± 62.6 vs. 50.9 ± 21.1 ng/ml, respectively, p = 0.032, Fig. 4A). Cd effects on IL-6 production by RA synoviocytes were rather like those in PVNS. At 0.01 and 0.1 ppm, Cd had no significant effect on IL-6 secretion compared to the control, although a slight decrease was observed with 0.1 ppm (83.3 ± 70.6 ng/ml vs. 93.3 ± 62.6 ng/ml, Fig. 4A). At 1 ppm and even more so at 10 ppm, IL-6 secretion was strongly decreased compared to the control (47.0 ± 28.3 and 37.4 ± 22.9 ng/ml respectively, vs. 93.3 ± 62.6 ng/ml, p = 0.001, Fig. 4A).

The kinetics experiment revealed that the Cd effect on IL-6 appeared at 24 h for PVNS cells but was significant at 6 days for both PVNS and RA cells (p = 0.009, two-way ANOVA test, Fig. 4B). In one RA patient, at 6 h, Cd increased IL-6 production and then started to decrease it at 72 h (Fig. 4B).

In both PVNS and RA synoviocytes, IL-6 production was decreased in the presence of Cd, probably because of cell death. Nevertheless, this does not exclude the possibility of other mechanisms that will require further studies. The Cd effect seemed to start at a lower concentration for RA compared to PVNS synoviocytes, reinforcing the conclusion that PVNS synoviocytes are less sensitive to Cd than RA synoviocytes.

### A pro-inflammatory environment increases the cytotoxic effects of Cd

**Figure 5:**
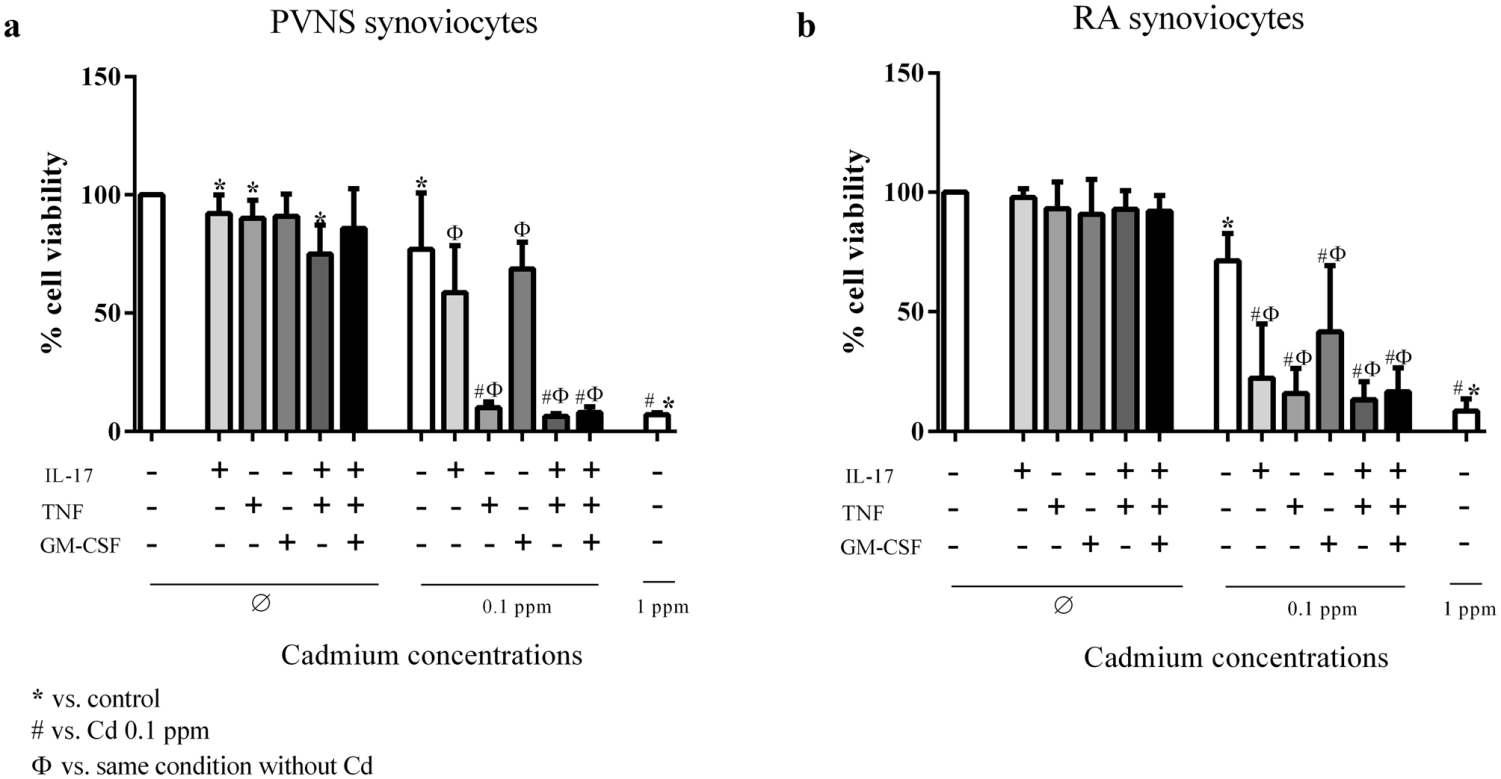
Quantification of cell viability of PVNS and RA synoviocytes 6 days after Cd exposure. PVNS (**a**) and RA (**b**) synoviocytes, used between passages 4 to 9, were treated or not with 0.01 ppm of Cd, in the presence or the absence of pro-inflammatory cytokines (IL-17: 50 ng/ml; TNF: 1 ng/ml; GM-CSF: 10 ng/ml). Cell viability was quantified by neutral red assay of PVNS (**a**) and RA (**b**) synoviocytes. Results are presented as mean ± SD, *p < 0.05. n = 6 from 3 PVNS patients and 3 RA patients.

A long list of cytokines such as TNF, IL-17, GM-CSF are involved in joint destruction as in RA and other joint diseases^15^. In addition, as demonstrated in RA synoviocytes, the addition of pro-inflammatory cytokines increased the sensitivity of RA synoviocytes to Cd-induced cell death^12^. Following this observation, PVNS and RA synoviocytes were exposed to inflammatory cytokines, IL-17 (50 ng/ml), TNF (1 ng/ml) and GM-CSF (10 ng/ml), alone or in combinations, to evaluate their effect on Cd sensitivity. Cell viability was measured by neutral red assay, 6 days after the addition of Cd treatment at 0.1 ppm (Fig. 5).

In the absence of Cd, IL-17, TNF or GM-CSF alone induced a 10% decrease of cell viability compared to the control in PVNS synoviocytes (IL-17: 92.1 ± 7.9%, p = 0.031; TNF: 90.1 ± 7.6%, p = 0.016; GM-CSF: 90.9 ± 9.4%, p ≥ 0.05, vs. 100% for control, Fig. 5a). The combination of TNF and IL-17 decreased even more cell viability (IL-17/TNF: 74.9 ± 12.3% vs. 100%, p = 0.031, Fig. 5a) but this effect was limited with GM-CSF (85.9 ± 16.7% vs. 100%, p ≥ 0.05, Fig. 5a). For RA synoviocytes, cytokines alone did not significantly decrease cell viability (IL-17: 97.9 ± 3.7%; TNF: 93.2 ± 11.1%; GM-CSF: 90.9 ± 14.5%, vs. 100%, Fig. 5b) nor with cytokine combination (IL-17/TNF: 92.9 ± 7.8%; IL-17/TNF/GM-CSF: 92.1 ± 6.5% vs. 100%, Fig. 5b).

Addition of 0.1 ppm of Cd decreased cell viability of both cell types with a higher decrease for RA than PVNS synoviocytes (71.3 ± 11.5% vs. 76.9 ± 23.9% respectively, p = 0.031, Fig. 5). Raising the Cd concentration to 1 ppm strongly increased its cytotoxic effect, reducing by 90% cell viability in PVNS and RA synoviocytes (7.0 ± 0.9% vs. 8.6 ± 5.0% respectively, p = 0.031, Fig. 5).

At 0.1 ppm of Cd, the presence of IL-17 and GM-CSF did not significantly increase the Cd effect in PVNS synoviocytes compared to Cd alone (IL-17: 58.6 ± 20.0%, GM-CSF: 68.7 ± 11.2%, vs. 76.9 ± 23.9%, Fig. 5a) while TNF strongly decreased cell viability (10.1 ± 2.2% vs. 76.9 ± 23.9%, p = 0.016, Fig. 5a). Conversely, in RA cells, the presence of these cytokines in addition to Cd, strongly decreased cell viability compared to Cd 0.1 ppm (IL-17: 22.2 ± 22.8%, GM-CSF: 41.5 ± 27.8%, TNF: 15.8 ± 10.5%, vs. 71.3 ± 11.5%, p = 0.031, Fig. 5b). The combination of cytokines had no stronger effect than TNF alone in both PVNS and RA synoviocytes (PVNS cells: TNF/IL-17: 6.4 ± 1.0%, TNF/IL-17/GM-CSF: 8.1 ± 2.3% vs. 10.1 ± 2.2% for TNF alone; RA cells: TNF/IL-17: 13.2 ± 7.6%, TNF/IL-17/GM-CSF: 16.4 ± 10.3% vs. 15.8 ± 10.5% for TNF alone, Fig. 5). In the presence of 0.1 ppm of Cd, addition of TNF led to a similar effect on cell viability to exposure with Cd at 1 ppm alone for both PVNS and RA synoviocytes (7.0 ± 0.9% and 8.6 ± 5.0% respectively, p = 0.031, Fig. 5).

These results confirmed that Cd-induced cell death was strongly increased in an inflammatory environment, mainly through the effect of TNF.

### Cd exposure reduces IL-6 production by PVNS synovial tissue

**Figure 6:**
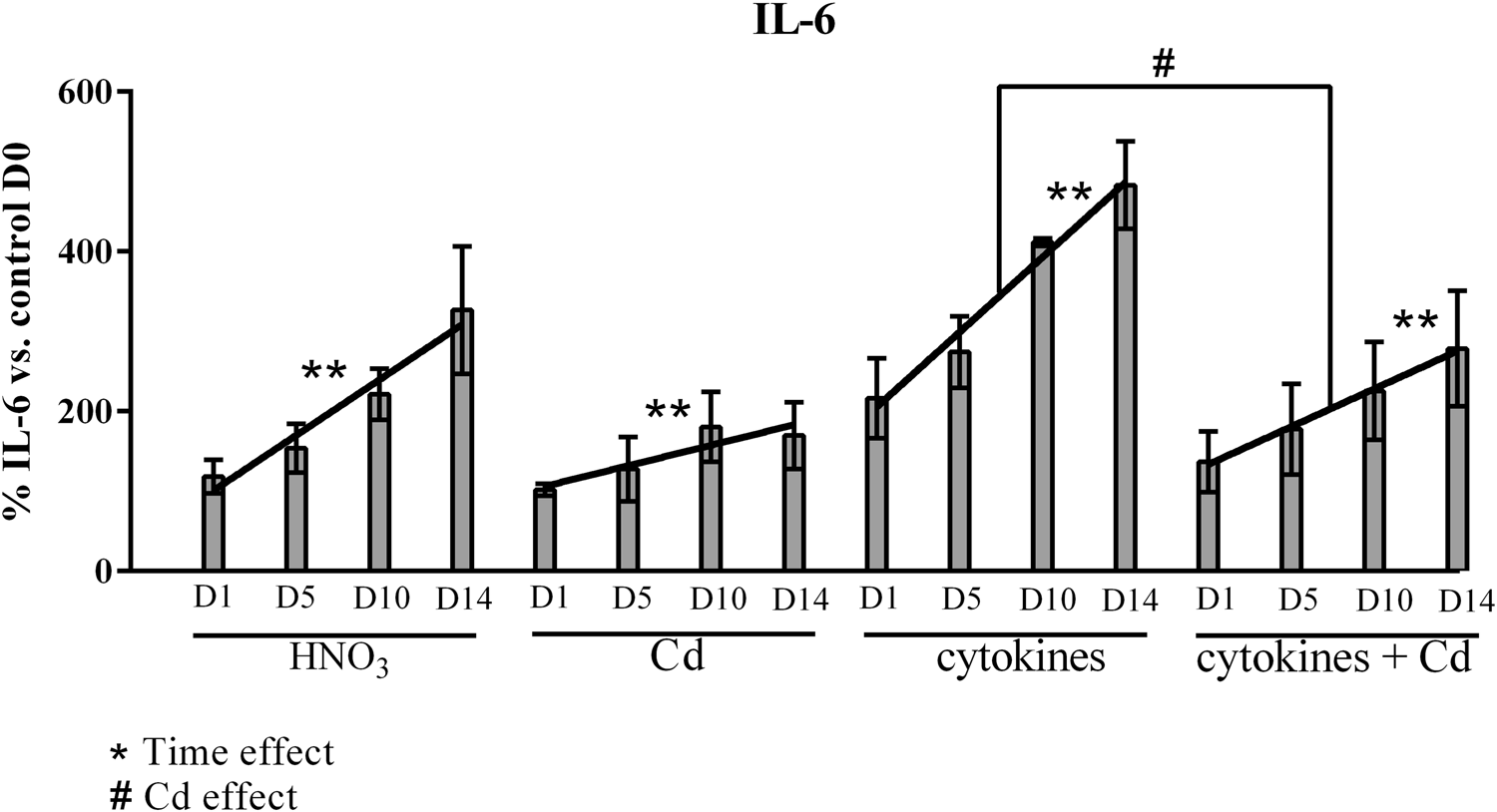
IL-6 production in supernatants from PVNS synovial tissue at different timepoints after Cd exposure. Supernatants from PVNS synovial tissues from three patients were collected at day 0, 1, 5, 10 and 14 after the treatment with 1 ppm of Cd with or without addition of the combination of pro-inflammatory cytokines (IL-17: 50 ng/ml and TNF: 1 ng/ml). IL-6 production was quantified by ELISA. Results are presented as mean ± SD. n = 3 from 3 PVNS synovial tissues.

To corroborate these observations with the clinical situation, explants of PVNS synovial tissue were treated with 1 ppm of Cd, in the presence or the absence of pro-inflammatory cytokines, IL-17 (50 ng/ml) and TNF (1 ng/ml), in combination. After different durations of exposure (D1, D5, D10 and D14), supernatants were recovered to quantify the production of IL-6. IL-6 concentrations at D1, D5, D10 and D14 were expressed as a percentage compared to the concentration at day 0, (D0), which is before the treatment and used as 100% control (Fig. 6). As described above, this cytokine is associated with the physiopathology of PVNS and RA and it has also been demonstrated that the reduction of IL-6 levels was correlated with the amount of Cd in cultures of RA biopsies^12^.

Without Cd, spontaneous IL-6 production increased with time, and was further upregulated by the presence of exogenous cytokines (Fig. 6). A two-way Anova analysis showed that IL-6 concentration increased over time, as expected (without cytokines: p = 0.008; with cytokines: p = 0.004, Fig. 6). In the presence of Cd, this increase was reduced and this reduction was significant in the presence of cytokines (p = 0.01, Fig. 6), probably because of increased cell death.

In conclusion, pro-inflammatory cytokines reinforced the effect of Cd on PVNS biopsy explants, reflecting the conditions found in an active PVNS joint.

### Cd exposure differently regulates the expression of modulators and exporters of Cd in PVNS vs. RA synoviocytes

To explain the increased sensitivity to Cd in an inflammatory context, observed with both synoviocytes and synovial tissue, the regulation of the pathways associated with Cd cell entry was analyzed by quantitative real-time PCR of gene expression of Cd transporters and modulators. Cd shares its cellular transporters with zinc. ZnT1 is the first described mammalian zinc transporter, the most ubiquitously expressed and the only member that predominantly functions on the plasma membrane as a zinc exporter^16–19^, contributing to zinc resistance^20^. ZIP-8 is important for the uptake of Cd^21^ and plays a significant role in Cd toxicity^22^. MT-1X, MT-1M and MT-1F are metallothioneins that control metal homeostasis in cells, contributing to heavy metal detoxification and these MTs bind to Cd tightly^23–25^. These data led us to study the expression of these transporters and modulators.

**Figure 7:**
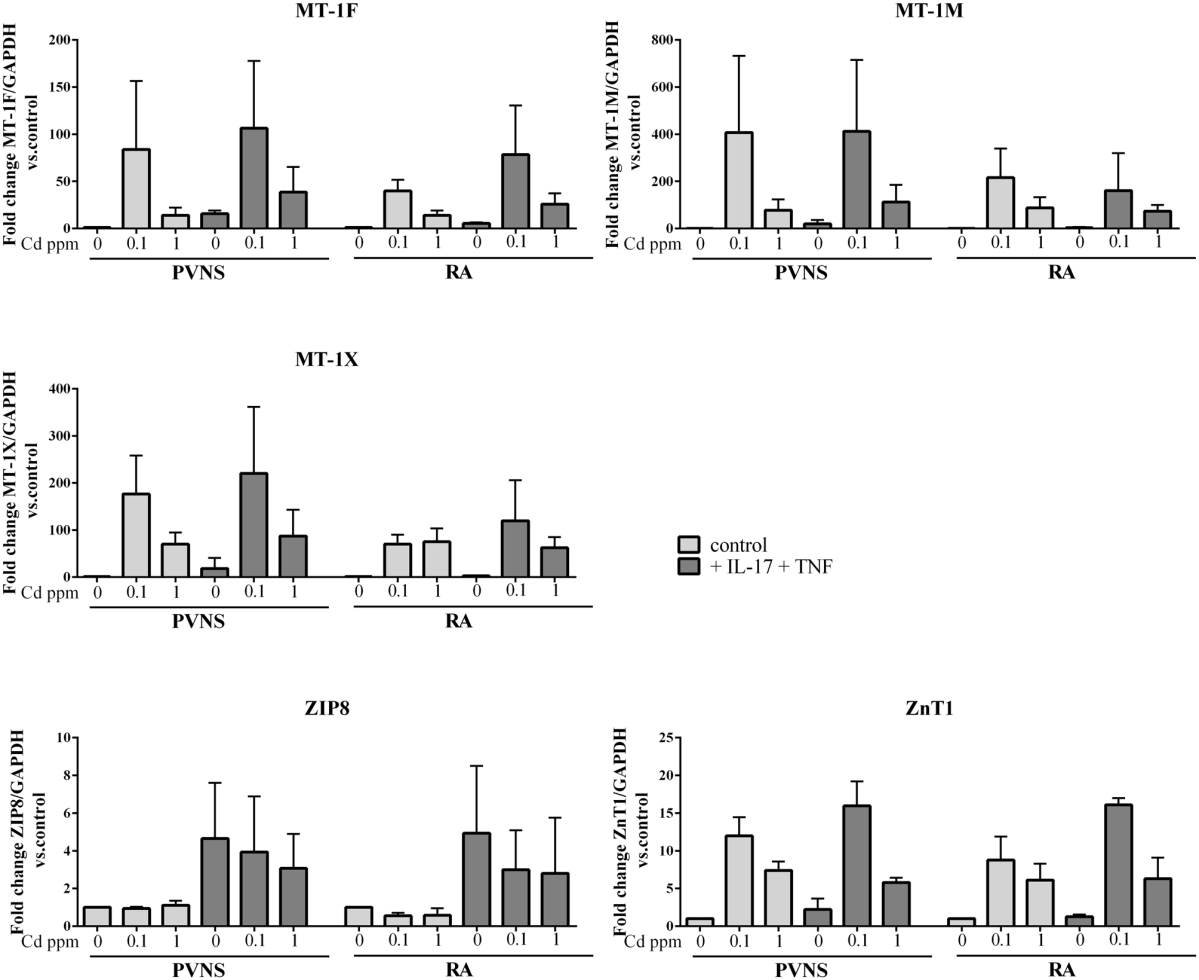
Gene expression of Cd transporters and modulators in PVNS and RA synoviocytes 6 h after Cd exposure. MT-1F, MT-1M, MT-1X, ZIP-8 and ZnT1 mRNA expressions were quantified by quantitative real-time PCR in PVNS and RA synoviocytes treated overnight or not with pro-inflammatory cytokines IL-17 (50 ng/ml) and TNF (1 ng/ml) or in combination and exposed to Cd at 0.1 and 1 ppm. The quantification was done 6 h after Cd exposure. Results are presented as mean ± SD. n = 3 from 3 PVNS patients and 3 RA patients.

PVNS and RA synoviocytes were treated with different Cd concentrations (0.1 or 1 ppm) associated or not with the pro-inflammatory cytokines IL-17 (50 ng/ml) and TNF (1 ng/ml), or their combination. After 6 h of Cd treatment, RNA was extracted and treated for RT-qPCR. mRNA expression of Cd transporters and modulators (ZIP-8, MT-1X, MT-1M, MT-1F and ZnT1) was then quantified in PVNS and RA synoviocytes and compared in fold change vs. control condition, normalized at 1 (Fig. 7).

Regarding the expression of the importer ZIP-8, Cd had no effect in PVNS synoviocytes (Cd 0.1 ppm: 0.9 ± 0.1 fold; Cd 1 ppm: 1.1 ± 0.2 fold, Fig. 7) while Cd decreased ZIP-8 expression in RA synoviocytes compared to the control (Cd 0.1 ppm: 0.6 ± 0.2 fold; Cd 1 ppm: 0.6 ± 0.4 fold, Fig. 7). The combination IL-17/TNF increased ZIP-8 expression of about fivefold in both PVNS and RA synoviocytes, compared to the control (4.7 ± 3.0 fold and 4.9 ± 3.6 fold, respectively, Fig. 7). In the presence of cytokines, Cd, at 0.1 or 1 ppm, decreased slightly ZIP-8 expression in both PVNS and RA synoviocytes compared to cytokines alone (PVNS: 3.9 ± 3.0 and 3.1 ± 1.8 fold, respectively, vs. 4.7 ± 3.0 fold, RA: 3.0 ± 2.1 and 2.8 ± 3.0 fold, respectively, vs. 4.9 ± 3.6, Fig. 7).

Regarding the expression of the exporter ZnT1, results were similar between PVNS and RA synoviocytes. Cd alone increased ZnT1 expression, mainly at 0.1 ppm (PVNS: Cd 0.1 ppm, 12.0 ± 2.5 fold; Cd 1 ppm, 7.4 ± 1.2 fold; RA: Cd 0.1 ppm, 8.8 ± 3.1 fold; Cd 1 ppm, 6.1 ± 2.2 fold, Fig. 7). Cytokine addition increased this effect only at Cd 0.1 ppm (PVNS: 16.0 ± 3.2 fold; RA: 16.1 ± 0.9 fold, Fig. 7).

The expression of the modulators MT-1F, MT-1 M and MT-1X increased after Cd exposure, mainly at 0.1 ppm, for both PVNS and RA synoviocytes but with a higher effect for PVNS cells (Fig. 7). The presence of cytokines had different effects depending on the modulator and the cell type. MT-1F expression was increased by the combination IL-17/TNF, in presence or not of Cd, for both PVNS and RA synoviocytes (Fig. 7). MT-1 M expression slightly decreased with cytokines in the presence of Cd in RA but not in PVNS synoviocytes (Fig. 7). For MT-1X, IL-17/TNF combination increased its expression in PVNS synoviocytes, with or without Cd (Fig. 7), while in RA, this increase was seen only in the control and at Cd 0.1 ppm (Fig. 7).

To conclude this part, modulators and transporters of Cd were rather similarly regulated in PVNS and RA synoviocytes. However, a stronger effect of Cd, mainly at 0.1 ppm, was observed in PVNS in comparison to RA synoviocytes. This difference of expression in Cd modulators and transporters in PVNS synoviocytes could contribute to their lower sensitivity to Cd-induced cell death, compared to RA cells.

## Discussion

Both PVNS and RA are articular diseases characterized by a hyperproliferation of synoviocytes leading to a hyperplasia of the synovium. In line with these observations, induction of apoptosis targeting directly synoviocytes could be an interesting treatment option for both diseases, even more so for PVNS, which is a single-joint disease.

Metals have been used for treatment in various indications, such as gold for arthritis or platinum for cancer. Although toxicity frequently was a problem, their efficacy justified their clinical use, until better treatments became available^26–28^. Intra-articular administration of treatment started, in RA, with steroids followed by radioactive compounds. Radioactive synovectomy has also been used in PVNS^29^, usually following surgery. However, the current limitations in the use of radioactive materials imply that there is today no medical intra-articular treatment for PVNS^30^, although this disease would be the perfect indication. Thus, there is a critical need for new therapeutic options for this disease.

The use of heavy metals has been considered as an interesting potential treatment option. Heavy metals, especially Zn, modulate immune responses^31,32^. Zn was found to modulate, in an opposite way of Cd, the inflammation and cell viability of RA synoviocytes^33^. Cd displays multiple roles in cell death^34^, notably by inducing apoptosis in several cell types as mesangial cells^35^ or neuronal cells^36^. Nevertheless, Cd-induced cell death could be beneficial if it follows mitogenic or mutagenic event. This beneficial aspect could be explored in chronic inflammation by inducing cell death of hyperproliferative cells, as synoviocytes in RA or PVNS. Previous results in RA models have shown that Zn addition enhanced IL-6 production^37^ but had no effect on synoviocyte viability^38^ while treatment with Cd induced massive cell death with anti-proliferative and anti-inflammatory effects on RA synoviocytes along with a reduction of IL-6 production^12^. Furthermore, Zn was able to reverse the effects of Cd^38^. In the context of PVNS, the anti-proliferative and anti-inflammatory effects of Cd first shown on RA synoviocytes were of particular interest because of the hyperplastic characteristics of the disease. Thus, the current results have showed these effects on PVNS synoviocytes compared to those on RA synoviocytes.

Observation by light microscopy showed a massive cell death effect induced by Cd exposure on PVNS synoviocytes. This was associated with a reduced cell viability starting with 1 ppm of Cd, at least in part through increased apoptosis, reflected by an increased Annexin-V staining. The mechanism of apoptosis was also tested by an ELISA of cleaved caspase 3. However, this assay lacks sensitivity with slow-growing synoviocytes. The results showed an increase of cleaved caspase 3 at 72 h in the presence of Cd 1 ppm (data not shown). Reduced cell number was correlated with reduced IL-6 production. These results were in line with those obtained with RA synoviocytes. The key difference was a lower sensitivity to Cd of PVNS vs. RA synoviocytes. On average, there was a one-log difference, with Cd-induced cell death starting with 0.1 ppm for RA synoviocytes vs. 1 ppm for PVNS synoviocytes.

This lower sensitivity could be explained in part by differences in the pathogenesis between RA and PVNS. PVNS was first considered as an inflammatory disease with many similarities with RA, including joint destruction as an endpoint. More recently, the identification of molecular translocations has linked PVNS with cancer^39^. The reduced sensitivity to cell-death inducing signals is, thus, in line with PVNS being at the border between a chronic inflammatory disease and a tumor disease. Additional studies could be performed with sarcoma cells, and it would make sense to detect a further reduced sensitivity to Cd.

An inflammatory environment is found in both RA and PVNS. With treatment application in mind, it is important to understand if inflammation would have a positive or negative effect on Cd activity. In a previous study, the sensitivity of RA synoviocytes to Cd-induced cell death was increased in an inflammatory context obtained by an addition of combined TNF and IL-17. Furthermore, GM-CSF has been involved in RA synoviocyte proliferation^40^, and its receptor is expressed by numerous cells, including fibroblasts^41,42^. Thus, PVNS synoviocytes were exposed in the presence or the absence of Cd to the best-defined inflammatory cytokines (IL-17, TNF and GM-CSF) used alone or combined, as would be the case at the disease site. Out of these cytokines, mainly TNF had a major role in Cd sensitivity. The presence of this cytokine allowed a decrease by tenfold in the Cd concentration needed to obtain the same effect in the absence of cytokines. Similar results were obtained with RA synoviocytes, in agreement with previous results^12^. These results confirm and extend the major role of TNF in Cd sensitivity of synoviocytes, already highlighted in RA^43^, and now in PVNS.

In mammalian cells, many proteins has the capacity to bind Zn, but three classes are the main regulators of Zn storage, compartmentalization and transport: Zn transporters (ZnT), Zn importers (ZIP) and metallothioneins (MT). ZnT family regulates the efflux of Zn from the cytosol while ZIP family regulates Zn transport in the opposite way. MT family can bind avidly to Zn to facilitate storage or metal detoxification^18,19,25^. Due to its high hydrophilicity, Cd needs active or passive transport proteins to enter cells. Zn and Cd share the same transporters, which are expressed by fibroblasts, notably the importer ZIP-8^21^ and the same modulators inside the cell, the metallothioneins MTs-1^23,24^. Cd is even preferentially absorbed by synoviocytes compared to Zn^38^. Our previous studies have also shown that the inflammatory context plays a critical role in the regulation of Zn transporters and modulators. Inflammatory cytokines increased ZIP-8 and MTs-1^12,37^, increasing intracellular uptake and storage of Cd in synoviocytes^12^. This regulation by inflammation could result in a stronger effect of Cd in inflammatory context. The lower sensitivity of PVNS compared to RA cells could be related in part to a different regulation of transporters and modulators of Cd. Expression of importers and exporters of Cd was rather similarly regulated in PVNS and RA synoviocytes. As previously described, the presence of IL-17 and TNF increased the expression of ZIP-8, the most important importer of Cd, in both PVNS and RA synoviocytes. This increase was in line with the highest sensitivity of cells to Cd in an inflammatory environment as cytokine exposure increased Cd uptake and accumulation into cells, which was not reversible, reflected by the absence of excreted Cd in the medium after washing^12^. An important difference was observed for metallothioneins that control metal homeostasis in cells^23,24,44^. In fact, MT-1F, MT-1M and MT-1X expression was higher in PVNS than in RA synoviocytes in both control and inflammatory conditions. Such changes could explain why PVNS cells are less sensitive to Cd than RA cells. Nevertheless, more investigations are needed to confirm the role of Cd transporters and modulators in its mechanism of action and in the different sensitivity to Cd between PVNS and RA cells.

To corroborate these results with the disease situation, PVNS synovial tissue was directly treated with increasing concentrations of Cd in the presence or absence of pro-inflammatory cytokines. As already shown with RA biopsies^12^, Cd induced a decrease of IL-6 production by PVNS synovial tissue, and this effect was enhanced by the presence of pro-inflammatory cytokines.

To extend the in vitro effects of Cd exposure, previous studies have developed an in vivo RA model, the Adjuvant-Induced Arthritis (AIA) rat model^12^. The intra-articular Cd-injection in AIA rats showed a protective effect on both synovitis and joint destruction, without systemic spread or hepatotoxicity^12^. Based on the results obtained in vitro in PVNS, it would seem logical to extend these to an in vivo PVNS model. However, the main limitation with PVNS was the lack of adequate experimental models. Various experimental attempts have failed to reproduce PVNS lesions for long term studies^45^. The development of a xenograft model allowed to establish a PVNS model by the transplantation of primary human tumor sample under the renal capsule of NOD SCID mice^46^. More work is needed to develop an in vivo PVNS model that could allow the evaluation of new options of PVNS treatment.

Extension of these Cd studies to the human situation is obviously a long-term goal. The toxicity of Cd could be a limitation notably by interfering with the homeostasis of essential metals^47^. It was suggested that Cd could contribute to RA pathogenesis^11^, notably because Cd inhalation could cause a form of nodular RA^48^. Nevertheless, the efficacy and toxicity of intra-articular administration of Cd have been evaluated in an arthritis rat model^12^. The injection of Cd reduced inflammation and protected the joint from destruction. It also proved to be safe as no cutaneous inflammation was detected at the injection site. The effect of Cd on the liver was also evaluated. For an injection of 0.1 and 1 ppm, no Cd was detected in liver samples. Only 0.1 ppm was detected after an injection of 10 ppm of Cd^12^. In addition, the levels of aspartate transaminases, used as an indicator for damage, remained unchanged regardless of the Cd concentration. Cd treatment of human hepatocytes did not affect their viability^12^. These results highlighted a positive benefit/risk profile for an injection of 1 ppm of Cd. In addition, intra-articular injection should greatly limit systemic diffusion.

Intra-articular injection of Cd represents a local exposure with limited risks to diffusion and thus limited risks to develop nodular RA. Since PVNS is a local disease, with usually no diffusion from the joint, this is the best indication for an intra-articular treatment. New modalities such as Cd-based quantum dots (CdTe-QDs) have been explored to further limit the possible Cd diffusion^49^. Other strategies have been also tested in our laboratory to target synoviocyte hyperproliferation. These include the use of a pro-apoptotic gene PUMA into RA synoviocytes and its induction in the AIA model has already shown positive results^50^.

In conclusion, Cd induced cell death and reduced inflammation in PVNS synoviocytes and synovium explants. Cd sensitivity of PVNS cells was lower than that of RA synoviocytes. This difference could be linked in part with a higher regulation of metallothioneins in PVNS cells. Use of Cd could represent a new therapeutic approach in inflammatory arthritic diseases. However, to extend this strategy to human joint diseases, new modalities need to be developed to improve the Cd treatment and limit the risk of systemic effects.

## Methods

All methods were carried out in accordance with relevant guidelines and regulations.

### Samples

Synoviocytes were obtained from patients undergoing joint surgery (Hospitals Edouard Herriot and Croix Rousse, Lyon, France), with diagnosed PVNS (mean age: 37 years, ratio F/M: 2/1) or with RA (mean age: 62 years, ratio F/M: 5/1), who fulfilled the American College of Rheumatology criteria for RA^51^. The synovial tissue was cut into small pieces and then adhered in 6 well-plates in Dulbecco’s modified Eagle’s medium (DMEM; Eurobio, Courtaboeuf, France) supplemented with 10% fetal bovine serum (FBS; Life Technologies, by Thermo Fisher Scientific, Carlsbad, USA), 2 mM l-glutamine (Eurobio) and 100 U/ml penicillin/streptomycin (Eurobio) (Complete DMEM). Cells were maintained at 37 °C in a humidified 5% carbon dioxide incubator and used between passages 4 to 9. Each patient signed an informed consent form. The protocol was approved by the Ethics Committee of the Hospitals of Lyon for the protection of persons participating in biomedical research under the number AC-2016-272.

### Biopsy assay

The synovial tissue was cut into small pieces of similar size and put into 24-well plates for adherence in complete DMEM. After 1 day of incubation, biopsies were treated with pro-inflammatory cytokines IL-17 (50 ng/ml, DDX-C-rhuIL17A, Dendritics, Lyon, France) and TNF (1 ng/ml, 210-TA, R&D systems, Minneapolis, USA), alone or in combination, as previously described^12^. One day after cytokine’s treatment, supernatants were recovered (Day 0) for normalization and biopsies were exposed to Cd at 1 ppm (UMR 5276, University of Lyon, Lyon, France). Then, supernatants were collected at days 1, 5, 10 and 14 for further analysis of IL-6 production.

### Cytokine exposure

PVNS and RA synoviocytes were exposed to pro-inflammatory cytokines with concentrations already tested and approved on RA synoviocytes in previous studies: IL-17 (50 ng/ml, Dendritics); TNF (1 ng/ml, R&D systems); GM-CSF (10 ng/ml, 215-GM, R&D systems). Cytokines were added, alone or in combination, 24 h before the Cd treatment of PVNS and RA synoviocytes, as already described.

### Cadmium exposure

Cd nitrate stock solutions were provided by the geology laboratory of the University of Lyon (UMR 5276). Cd dilutions were done with HNO3. The amount of nitrate added to cultures as control was the same to that found in a 10 ppm of Cd condition. Results with and without HNO_3_ alone were similar, thus the results presented were mainly with HNO_3_. Untreated represented untreated cells and control represented cells treated with HNO_3_.

PVNS or RA synoviocytes were seeded in 24-well plates at a density of 7.5 × 10^4^ cells/well, overnight, in complete DMEM. The next day, synoviocytes were treated or not with Cd at different concentrations (0.01, 0.1, 1 and 10 ppm) and then incubated at different timepoints according to the different experiments.

### Light microscopy

At different timepoints after the Cd exposure (24 h, 72 h and 6 days), PVNS and RA synoviocytes were directly analyzed qualitatively by imaging using an epifluorescence Nikon Eclipse inverted microscope. Data were then analyzed using ImageJ software. Measured characteristics were the round morphology of cells, cell detachment, and cytoplasmic retraction.

### Annexin V staining

After 6 days of exposure to Cd, PVNS and RA synoviocytes were recovered and stained with Annexin V conjugated to fluorescein (FITC-Annexin V, ThermoFisher scientific, Waltham, Massachusetts, USA) at 3 µL per 100 µL of each cell suspension in the provided 1× annexin-binding buffer. After a 20 min incubation at room temperature, fluorescence emission was measured by flow cytometry at 530 nm (e.g., FL1, Navios, Beckman Coulter, Brea, California, USA). Cell population was separated depending on the level of fluorescence, live cells with a low level of fluorescence and apoptotic cells with a high fluorescence level. A negative control consisted of synoviocytes without Annexin V staining. For the kinetics studies, cells were recovered by trypsin after 6 h, 24 h, 72 h or 6 days and stained with Annexin V-APC (APC-Annexin V detection kit, ThermoFisher Scientific), according to the manufacturer’s instructions. After a 15 min incubation at room temperature, cells were washed with binding buffer and resuspended in 200 μL of binding buffer. 5 μL of Propidium Iodide (PI) was added in each tube and cells were analyzed by flow cytometry (Navios, Beckman Coulter).

### Cell viability

After 6 h, 24 h, 72 h or 6 days of exposure to Cd, PVNS and RA synoviocytes were assessed by Neutral red assay. Supernatants were removed and cells were incubated during 150 min with a neutral red solution diluted at 1/42 in DMEM, at a final pH of 6.5 (Sigma-Aldrich, St. Louis, M, USA). After centrifugation, cells were washed with PBS 1× and treated with acetic acid solution (1% of acid acetic in a solution of 50% ethanol and 49% of water; 450 µl/well). Absorbance was measured at a wavelength of 540 nm and 620 nm.

### Enzyme-Linked Immunosorbent Assays (ELISA)

IL-6 production was evaluated in culture supernatants with commercially available ELISA kit (Diaclone, Besançon, France), according to the manufacturer’s instructions.

### Gene expression of Cd transporters and modulators by quantitative real-time PCR

PVNS and RA synoviocytes (2.5 × 10^5^ cells/well, 6-well plates) were exposed, overnight, to pro-inflammatory cytokines IL-17 (50 ng/ml Dendritics) and TNF (1 ng/ml, R&D systems), or in combination. Then, cells were exposed or not to 0.1 or 1 ppm of Cd. After 6 h of Cd treatment, complete RNA was extracted using the RNeasy Mini Kit (Qiagen, Hilden, Germany) and quantified with the Quant-it kit assay (Invitrogenby Thermo Fisher Scientific, Grand Island, NY, USA) following manufacturer’s instructions. cDNA was synthesized using the QuantiTect reverse transcription kit (Qiagen) according to the manufacturer’s instructions. SYBR green-based real time qRT-PCRs were performed on the CFX96 Real-Time PCR Detection System (BioRad, Hercules, CA, USA) using the QuantiFast SYBR green kit and QuantiTect primers (Qiagen, ZIP-8: QT01885583; ZnT1: QT00096320; MT-1F: QT01004605; MT-1M: QT01004626; MT-1X: QT00048237). Cycle threshold values were normalized with respect to the endogenous control gene glyceraldehyde 3-phosphate dehydrogenase (GAPDH: QT01192646). The relative expression of ZIP-8, ZnT1, MT-1F, MT-1M and MT-1X genes was determined using the comparative threshold cycle method and expressed in fold change compared to the control condition (without cytokines and without Cd).

### Statistical analysis

Statistical analyses were performed using paired Wilcoxon test or a two-way ANOVA for the kinetics experiments and Fig. 6. All analyses were performed with GraphPad Prism 5 software. *p* values less than 0.05 were considered as significant.

### Ethical approval information

The protocol was approved by the Ethics Committee of the Hospitals of Lyon under the number AC-2016-272.

## Data Availability

All data relevant to the study are included in the article.
